# Virtual product development process for reducing noise, vibration, and harshness of vehicle based on substructuring and artificial neural network

**DOI:** 10.1038/s41598-022-16645-x

**Published:** 2022-07-28

**Authors:** Yongdae Kim, Un-Chang Jeong

**Affiliations:** 1 Hyundai Motors Company, 165-24, Hyundaiyeonguso-ro, Namyang-eup, Hwaseong-si, Gyeonggi-do Republic of Korea; 2 Department of Engineering, Smart Vehicle Engineering, Wonkwang University, 460, Iksan-daero, Iksan-si, Jeollabuk-do Republic of Korea

**Keywords:** Engineering, Mathematics and computing

## Abstract

In this study, virtual product development method for reducing vibration and noise is proposed for designing at the concept development stage of a vehicle. To this end, the vibration characteristics of the system are predicted through the Lagrange-multiplier frequency-based substructuring technique. The concepts of contact, blocked and transmitted force, and force transmissibility were used for determining the improvement subsystem or combination of subsystems when using the modular platform. Moreover, after the subsystems to be improved were determined, Artificial Neural Network was used as a method of predicting vibration characteristics according to the change of design variables. To verify this, the prediction of the blocked force was performed by changing the young’s modulus of the simplified substructure. Finally, the reduction in response was confirmed by applying the blocked force of the simplified subframe to the simplified structure, and a vehicle development process using a database at the concept setting stage is proposed.

## Introduction

A general vehicle development process can be summarized into three steps: concept-design-prototype testing. Engineers try to solve many problems in the concept development stage of a vehicle, but after the production of the prototype, many unexpected problems are identified. Thus, the engineers need to go back to the design stage again and try to solve the problems. Such a general development process is time-consuming and cumbersome, thereby increasing inefficiency and making it difficult to respond quickly to the market.

Studies have been conducted on the product development process to respond quickly to the market. Among them, the set-based design (SBD) process, which finds a new combination set of subsystem based on previous data, is representative.

SBD presents a series of alternatives instead of a single solution^1^. Through SBD, engineers determine and limit the range of the design variables^2^. A study in which SBD was applied to vehicle design by proposing these alternative sets was useful in the preliminary stages of detailed design^3^. Durward et al. claimed that if vehicle development teams and marketing teams participated in the development process in the manner of point-based serial design process, all organizing teams must review according to all changes, and there is no guarantee that the results would be converged^4^. However, in the proposed set-based concurrent engineering, assorted designs are proposed in advance, and the final draft is drawn out through collaboration with other teams. The proposed SBD method establishes a map of the performance space. In other words, from the perspective of full vehicles, each team determines the range of major performance. In addition, SBD builds feasible sets by integrating the space considered by each team. And SBD derives results by gradually narrowing the established sets through inter-team communication. Furthermore, uncertainty according to the process progress is controlled through these sets. However, this method makes it difficult to derive appropriate results if the vehicle’s performance map is tightly integrated.

In summary, the set-based design process is a method that is used in the concept stage based on previous data. Also, it is possible to proactively respond to the problems of the prototype testing stage since it helps the researchers know many problems in advance through preceding development. Moreover, it provides flexibility in the occurrence of issues in the development process. Recently, automobile manufacturers have established a modular platform commonization strategy by using the advantages of this set-based design process. In particular, Volkswagen and Toyota have continuously deployed new models and expanded their lineups to respond to customer needs in various regions, and at this time, they are pursuing a component commonization strategy to curb rising vehicle costs^5,6^. Volkswagen’s modular platform fixed specific sections in engine rooms with high design complexity and focused on applying standard parts. In terms of the fixed sections, the distance between the pedals in the front axle was fixed. Through this, it is easy to achieve significant cost reduction. Toyota’s modular platform fixed the length of the underbody and focused on applying standard parts within the underbody. It is characterized by standardizing large parts that have excellent commonization deployment effects such as engines, transmissions, and suspension parts. In order to maximize these advantages, it is necessary to convert the results of previously developed common parts into databases and use them in the preliminary stage of vehicle development. This is because if the performance of the product is preemptively predicted at the vehicle development concept stage, decision-making based on reasonable data is enabled, and time and effort to improve the product can be saved in the design stage.

In general, the process of predicting characteristics in the preceding stage of vehicle detailed design is called Virtual Product Development (VPD), and CAD/CAE are used. Starting with the development of the algorithm in the 1970s, it has been widely studied in CAD/CAE design, and various application software has been developed^7,8^.

VPD from a manufacturing and development perspective involves numerical analysis of 3D modeling. Substructuring, which is widely used among these analysis methods, is a technique that analyzes the system using dynamic characteristics of the structure. The advantage of the method is that it is modeled and analyzed in the most suitable domain. For example, it is possible to freely combine the experimentally measured results and analyzed dynamic characteristics. Moreover, models of various development groups can be shared and combined. Because the shared information requires only dynamic information (e.g. Frequency response function), the details (e.g. shape, material, and other security items) of the component model can be kept confidential. Therefore, it may be useful in joint development with partner companies^9^.

Substructuring methods include the modal synthesis method, component mode synthesis method, and frequency response function based synthesis (FBS) method. Among these, FBS is a method widely used in recent years; it uses transfer functions in the frequency domain. To predict characteristics with FBS, the transfer function matrices and modal information are required^10,11^.

The following should be considered to use the substructuring methods in modular platform strategy:

1. A strategy for the combination of subsystems are required. When combining subsystems, it should be possible to find an appropriate combination from the perspective of a vehicle through strategies. This is because the characteristics of subsystem may be changed by connection with the other subsystem.
2. A quantitative indicator to manage characteristics of subsystem in the database are needed. Since the characteristics of a subsystem change after the subsystem are connected to different subsystems, it is difficult to identify the characteristics unrelated to the connection. Therefore, it is necessary to find a quantitative indicator independent of the characteristics of the connection.
3. It is necessary to predict the characteristics of subsystems that are not in the database. When design variables are changed, it is necessary to predict the characteristics of the system based on experimental and analysis data. Previously, analysis has been performed on the number of all cases by changing design variables. However, to perform analysis on many subsystems in the database is time-consuming.

Methods for solving the aforementioned considerations are described below.

Since forces of the system is transmitted through connection parts, it is necessary to compare forces transmitted to the subsystem to select a combination set of subsystems that satisfy the target performance. Therefore, it is natural to use the contact force, which is the force generated at the connection, as an indicator of combination selection. The contact force is obtained using Lagrange-Multiplier-Frequency Based Substructuring (LM-FBS). LM-FBS is a technique that predicts the responses of the multi-level subsystems through a Boolean matrix containing connection information^12^.

General substructuring methods predict the characteristics of the system from the perspective of substructure impedance by first satisfying the compatibility condition of the displacement on the contact surface, then satisfying the equilibrium condition. However, this method directly obtains only the displacement between the contact surfaces. That is, LM-FBS establishes a formula to first satisfy the equilibrium condition from the perspective of substructure admittance, then the compatibility condition. LM-FBS has the advantage of directly obtaining contact force using a formula representing the magnitude of the contact force by the Lagrange Multiplier (LM), and easily performing assembly using a boolean matrix. Therefore, it is appropriately used for determining a combination of subsystems when developing a vehicle with a new concept based on data of various existing subsystems.

Moreover, it is necessary to manage the characteristics of subsystems in the database to combine subsystems based on the database, and because the characteristics change depending on the connection between subsystems, it is difficult to use the frequency response function or contact force. The blocked force, which has been widely studied in recent years, is a force that is independent of the characteristics of the connection to which subsystems are connected and is not subordinate to the interrelationship between the source and receiver^13^. It is used to predict the system’s response when the subsystem to which the exciting vibration is connected changes in the experiment. Therefore, the blocked force presents characteristics independent of connection; thus, it is suitable for the performance management of product in the database.

The characteristics of a subsystem that is not in the database is predicted through a numerical method. In general, in complex subsystems, numerical characteristics prediction has been performed through the sensitivity analysis of models by performing parametric model modification. Variables commonly used in parameter correction include mass, stiffness, and damping matrices. By using the orthogonal criterion and solving the model’s characteristic equation, Fox and Kapoor^14^ derived the sensitivity calculation equation and design parameters for the eigenvalues and eigenvectors of the linear structure. Thereafter, Rogers^15^ and Garg^16^ improved Fox’s research results and promoted the development of sensitivity methods. Nelson^17^ and Lim and Junkins^18^ simplified the sensitivity formula of feature vectors. However, model prediction by parametric or numerical study takes a lot of effort and time.

In recent years, researchers have increasingly preferred using artificial neural network (ANN) algorithms. Typical types of neural networks are feedforward neural networks, multilayer perceptron neural networks, and radial basic functional (RBF) neural networks. Pandey and Barari^19^ modified the model using a multilayer perceptron neural network and an error inversion algorithm. Atalla and Inman^20^ and Levin et al.^21^ modified models of one-dimensional frame structure and two-dimensional plate structure, respectively, through structural frequency response functions measured using the RBF neural network. ANNs are gradually expanding their scope of application^22^ and building a pretrained model has the advantage of taking less time than predicting using existing finite element methods when predicting new data. Therefore, it is necessary to examine the applicability of ANN to predict data that is not in the database when improving parts in the concept development stage of the vehicle development stage. If it is possible to learn using ANN and predict new improvement plans in advance, efficient improvement in product development time and effort is expected. If the improvement of subsystem is preemptively predicted in the concept stage from the perspective of vehicle characteristics, it is expected that the time and effort of detailed-level design and performance verification stage are reduced.

The rest of the paper is structured as follows. “Dynamic substructuring” predicts the vibration characteristics of the system through the substructuring technique. Through this, we discuss the need to use other concepts as a quantitative indicator when determining improvement parts or combinations of subsystem during the process of developing a vehicle. “Data-based virtual product development” section process in the concept development stage is proposed. Indicators of component combinations and subsystem management methods within databases are presented using contact force, transmitted force, blocked force, and force transmissibility. This Force transmissibility methodology has the advantage of being able to clearly compare the transmission of forces between subsystems rather than the existing contribution evaluation or transfer path analysis, making it easier to identify the improvement subsystem. Also, artificial neural network is proposed as a method of predicting the vibration characteristics and it is confirmed that the response was reduced by connecting improved subsystem to the original system, and a vehicle development process using a database at the concept setting stage is proposed. The prediction methodology using ANN presented in this study has the advantage of reducing time and effort compared to the existing prediction method using FEM. Therefore, this study is expected to contribute significantly to the study of virtual product development because it proposes a database-based product development method to reduce vehicle noise, vibration, and harshness at the concept stage.

## Dynamic substructuring

### Lagrange-multiplier FBS

Traditional substructuring methods were based on the dynamic equations of motion. LM-FBS, which has been widely studied recently, has similarly been derived based on the dynamic equations of motion and is easily combined with other models using the Boolean matrix. This section introduces LM-FBS as a representative substructuring technique and summarizes it.

**Figure 1:**
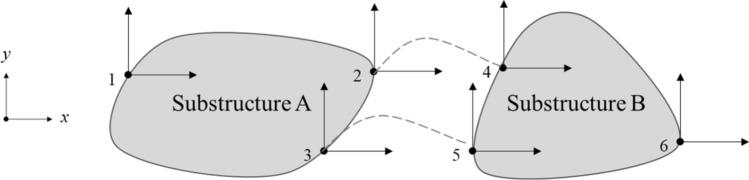
The system consisting of substructures A and B.

To explain this, Fig. 1 shows a system consisting of substructures A and B. The system is connected at two nodes, each of substructures A and B.

If the system is modeled in the frequency domain, it is presented as outlined in Eq. (1).

1 $${Z}^{\left(s\right)}\left(\upomega \right){u}^{\left(s\right)}\left(\upomega \right)={F}^{\left(s\right)}\left(\upomega \right)+{F}_{c}^{(s)}(\upomega )$$

where *Z*^(s)^(*ω*) is the system impedance, *u*^(s)^(*ω*) the node displacement, *F*^(s)^(*ω*) the external force, and *F*_*c*_^(s)^(*ω*) the contact force between connections.

The conditions of the system’s connection (interface compatibility) are summarized as Eq. (2) and expressed as a Boolean matrix containing connection information as Eq. (3).

2 $$\begin{aligned} {u}_{4}-{u}_{2} &=0 \\ {u}_{5}-{u}_{3}&=0\end{aligned}$$

3 $$Bu=0$$

where, *B* represents Boolean matrix containing connection information.

The contact force generated between the connection nodes was the force generated in the nodes connected between the substructures, and the connection characteristics of both substructures were reflected. If the contact force was expressed with a Boolean matrix containing connection information and *λ* (LM) representing magnitude, it was outlined as presented in Eq. (4).

4 $${F}_{c}=-{B}^{T}\lambda $$

Substituting Eq. (4) into Eq. (1), the following equation was obtained.

5 $${Z}^{\left(s\right)}\left(\omega \right){u}^{\left(s\right)}\left(\omega \right)+{B}^{T}\lambda ={F}^{\left(s\right)}\left(\omega \right)$$

If Eqs. (3) and (5) were expressed by matrix forms, the following equation was obtained.

6 $$\left[\begin{array}{cc}Z& {B}^{T}\\ B& 0\end{array}\right]\left[\begin{array}{c}u\\ \lambda \end{array}\right]=\left[\begin{array}{c}{F}_{c}\\ 0\end{array}\right]$$

Using admittance,

7 $$u=Y(f-{B}^{T}\lambda )$$

where, Y represents admittance matrix of system.

Multiplying both sides of Eq. (7) by Boolean matrix, the following equation was obtained.

8 $$Bu=BY(f-{B}^{T}\lambda )$$

When Eq. (8) was summarized with respect to λ and substituted into Eq. (7), an equation for an output node such as Eq. (9) was obtained.

9 $$u=\left[Y-Y{B}^{T}{\left(BY{B}^{T}\right)}^{-1}BY\right]f$$

Equation (9) shows that the response of the combined substructure is the result of subtracting the response related to the connection point from the response due to the external force of the unbound substructure. From the prerequisite that matrix *Y* of substructures and *B* containing connection information is known, the final response point can be calculated according to Eq. (9).

### Prediction of the vibration characteristics of the simplified structure

**Figure 2:**
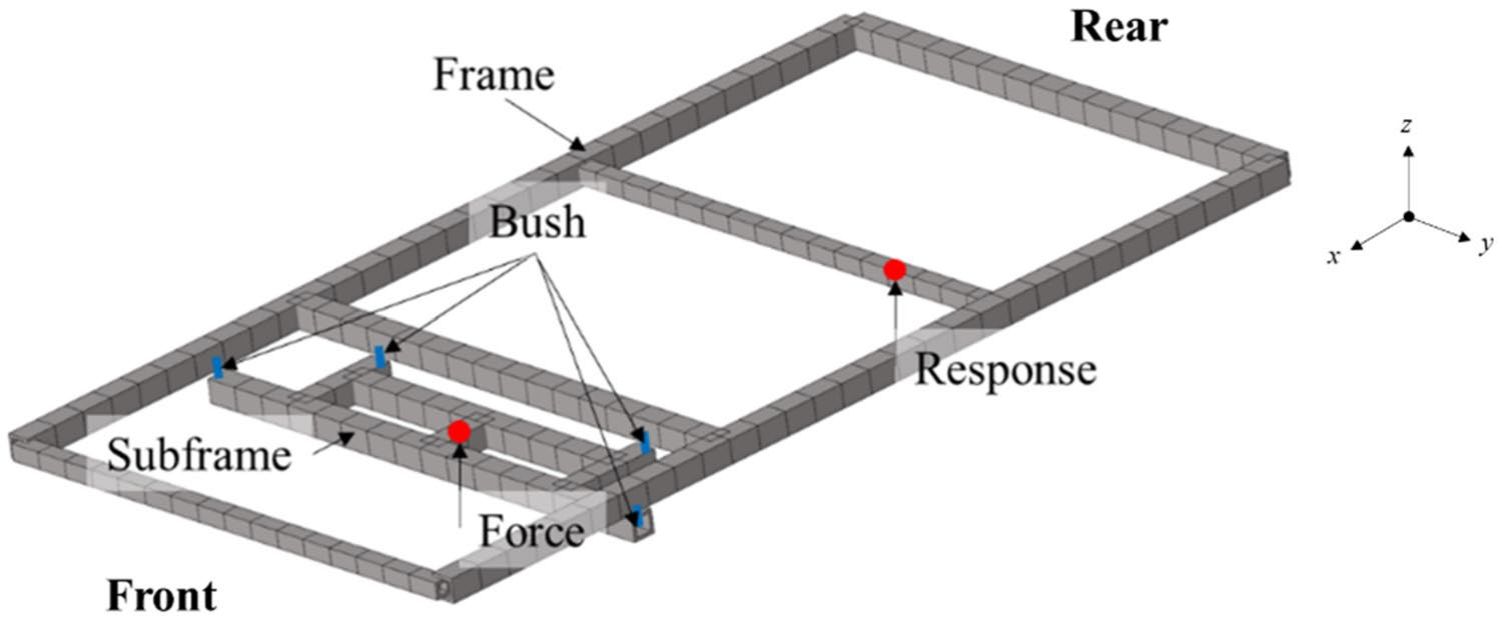
The simplified structure.

A vehicle is composed of tires, subframes, bushes, frames, and a body. The target of this paper are simplified subframes, bushes and frames, which are the main transmission paths of vehicle vibration. And one point on the subframe to which the force is transmitted and one point on the frame to which the response is calculated were selected as the responses (Fig. 2).

The bush characteristics of the simplified structure are as follows (Table 1).

**Table 1:** Bush properties of the simplified model.

Isolator	Stiffness (N/mm) in the following directions			Remark
	*x*	*Y*	*z*	
Front	4581	13,862	4836	C = 100 Ns/m
Rear	4719	13,009	3190	

**Figure 3:**
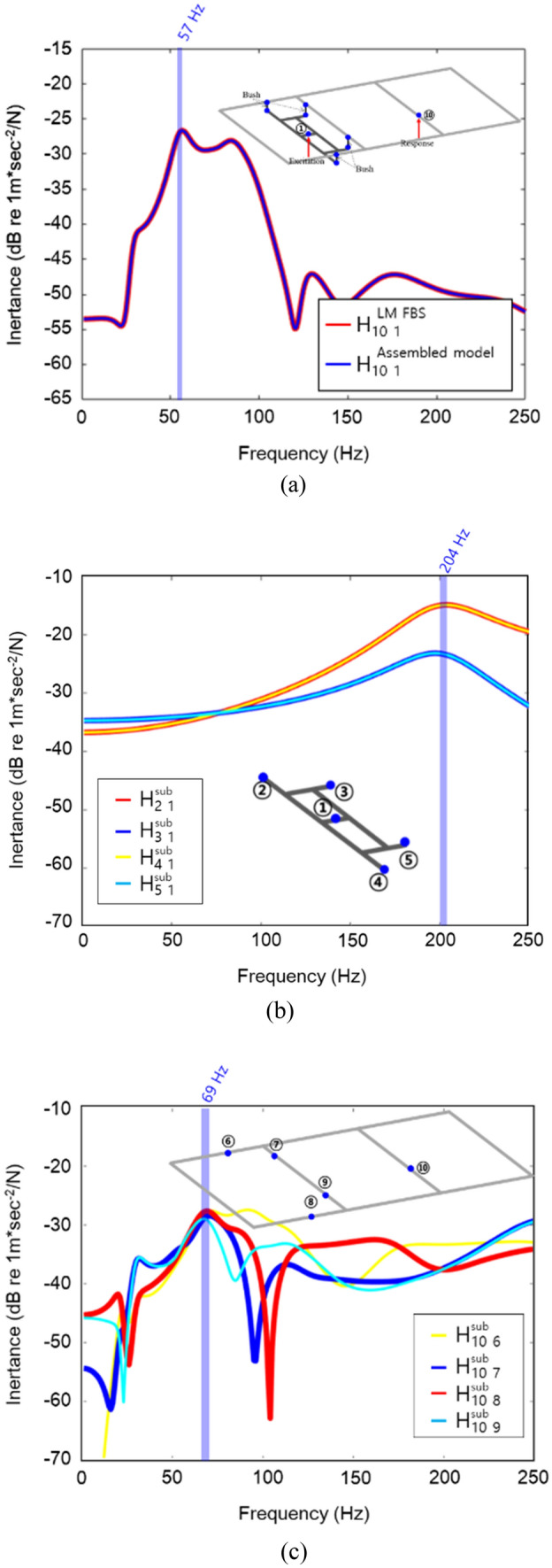
Frequency response functions. (**a**) The simplified structure; (**b**) Subframe; (**c**) Frame.

The response at the node on the frame was obtained using Eq. (9). Figure 3a shows the frequency response functions from the excitation point to response point. And Fig. 3b,c respectively show the frequency response functions from the excitation point of the subframe to the connection points and the frame connection points to the response point are presented.

Also, Fig. 3a shows the main natural frequency of the simplified structure was 57 Hz, and it was difficult to estimate the natural frequency of the simplified structure through the frequency response functions of subframe and frame. This was because when connecting subsystems, the characteristics of the subsystems changed due to the connection. For this reason, it was difficult to intuitively determine the subsystems that were problematic when combining subsystems in the concept development stage. Therefore, when developing a vehicle according to the modular platform strategy, it was necessary to use other concepts as an indicator when determining improvement subsystems or combinations of subsystems.

## Data-based virtual product development

It was discussed in the previous section that to find the appropriate combination of subsystems in the database, a strategy to deal with it was necessary. This section aims to present indicators for managing combinations and subsystems when developing virtual system through substructuring and to explain an example through the simplified model. Also, to perform predictions on subsystems in need of improvement, predictions were conducted using the ANN based on previously developed databases.

### Method of determining the appropriate combination of subsystems

**Figure 4:**
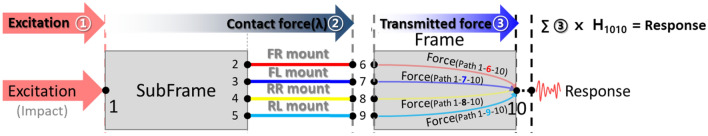
Schematic diagram of the power transmission process.

The excitation force applied to the subframe transmitted the subframe to the connection points of the bush, and these connection points excited the bush. And bush transmitted these forces to the connection points of the frame and passed through the frame and was transmitted to the response point, appearing as a response. Figure 4 schematically illustrates this process.

The force passing through the subframe, and the bush is transmitted to the frame, which is called a contact force. The contact force reflects the connection characteristics of the frame. The contact force was obtained using LM-FBS and the corresponding equation is presented in Eq. (10).

10 $${\lambda =({BYB}^{T})}^{-1}BY$$

In the equation of contact force, ${({BYB}^{T})}^{-1}$ represented the characteristics of the bush. Figure 4 presents the contact force calculated at four connection points on the frame.

**Figure 5:**
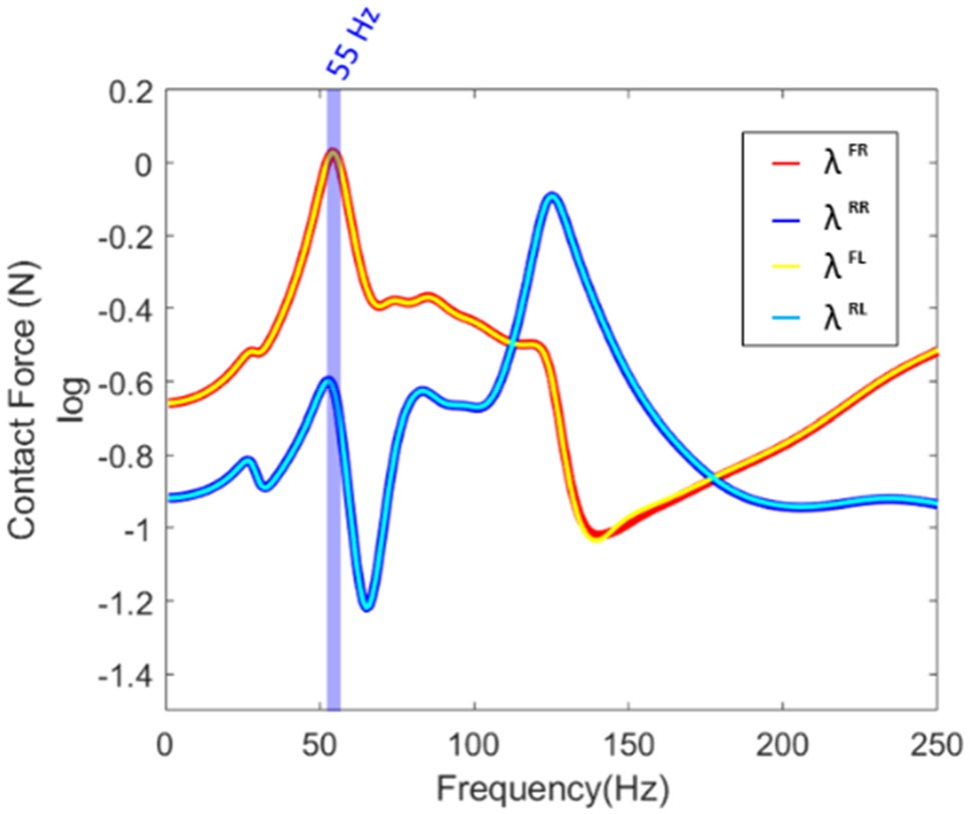
Contact forces (frequency domain).

Figure 5 shows, the contact force estimated the natural frequency of the connected system (Fig. 3a results). It is also possible to identify through which part the force was transmitted. For example, it was identified from the characteristics of 55 Hz that most of the force was transmitted through both sides of the front bush; hence, an improvement plan for subframe and bush were obtained.

The contact force transmitted to the frame delivered a force to the response node. Contact force could not be obtained because the response point was a free-end condition without a connected system. To identify the characteristics of this force at this node, the concept of transmitted force was introduced. This was because contact force was a force generated between two connected parts; thus, if the final response point was not connected to another part, a new concept was needed.

**Figure 6:**
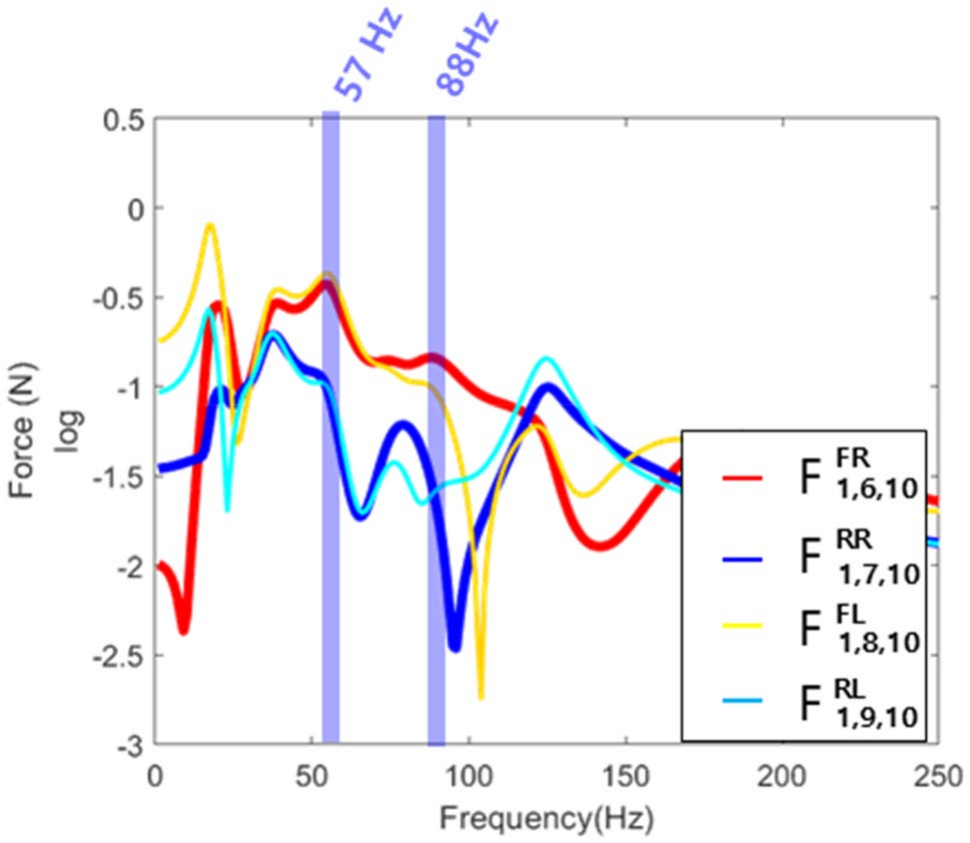
Transmitted force at the final response point.

In order to calculate the transmitted force, force transmissibility is need. Force transmissibility in multi-degree- of-freedom system has been derived by Lage et al.^23^. Using this, the formula of the transmitted force was expressed as Eq. (11).

11 $${T=({BYB}^{T})}^{-1}BY\times ({{H}_{ab}{{H}_{bb}}^{-1})}^{T}$$

where, H in ${H}_{ab}$ and ${H}_{bb}$ is frequency response function, *a* is the connection point between bush and frame, and *b* is the response point. Figure 6 shows the frequency characteristics of the transmitted force calculated using Eq. (11).

It was seen that the response of the response point at 57 Hz was attributed to the transmitted force of the front right and rear right paths. Therefore, to improve the vibration characteristics of the frame, it was necessary to establish countermeasures on the front right and rear right paths.

In summary, the main path of the system was found using the contact force at the connection part or the transmitted force at the response point. However, main subsystem for reducing vibration could not be found through comparison between forces. This was because the magnitude of the excitation force was reduced as it passed through the subsystem, making it difficult to directly compare the transmitted force of the response point with the contact force that had passed through the substructure. Therefore, the force transmissibility ratio was used to compare the contact forces and the transmitted forces for determining which part of the subsystem should be improved.

**Figure 7:**
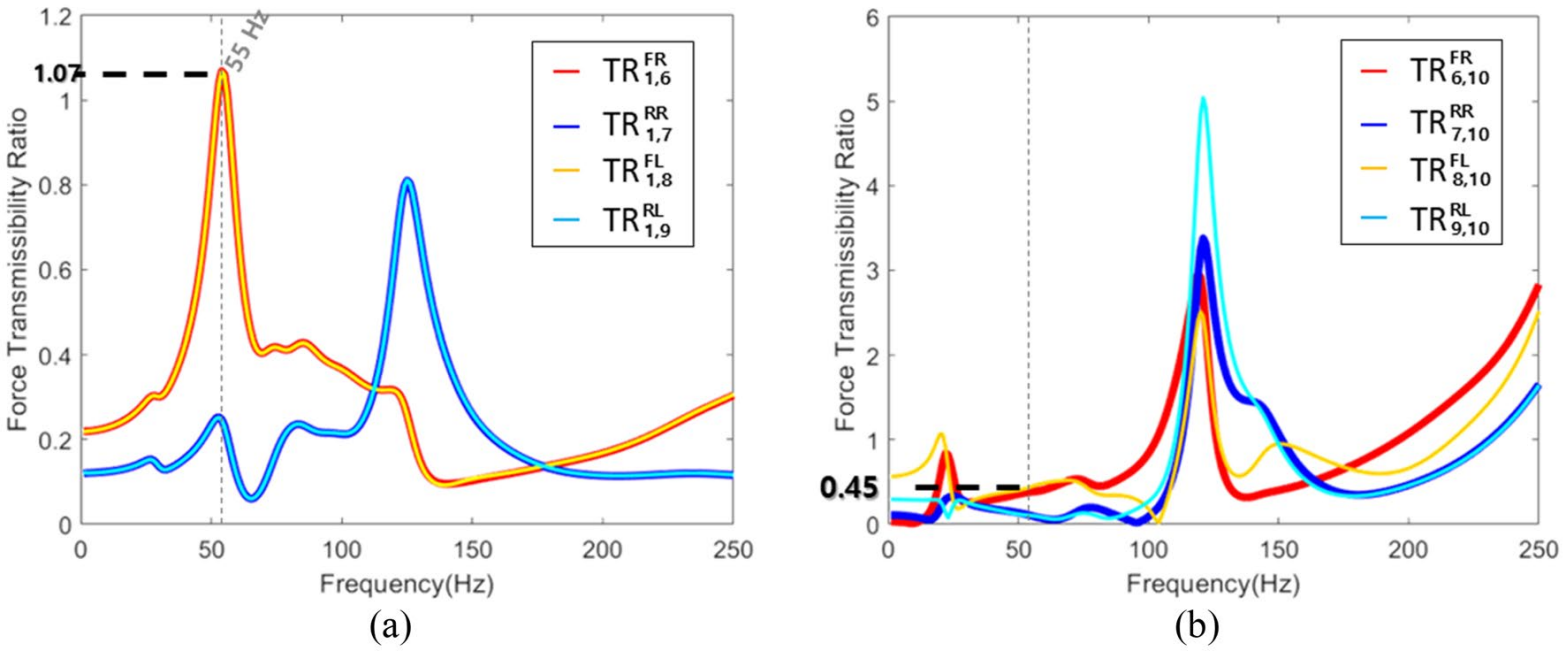
The force transmissibility ratio of the connection part and response point. (**a**) Connection part (using contact force); (**b**) Response point (using transmitted force).

In the case of contact force transmissibility ratio, impulse excitation became the input force. Because the excitation force applied to the subframe was impulse excitation. As well, for the transmitted force transmissibility ratio, the contact force became the input force. Figure 7 shows the equation for this force transmissibility ratio was presented in Eq. (12) and the results.

12 $$\text{Force} \; \text{ Transmissibility} \; \text{ Ratio}=\frac{{\uplambda}^{i}}{Impact \; Force}=\frac{\sum Transmitted \; Force}{{\uplambda}^{i}}$$

Through comparison of these two force transmissibility ratios, it was determined which part to improve or replace. For example, at 127 Hz, since the transmissibility ratios of the transmitted force was large, it was necessary to improve the frame. On the contrary, it was found that it was effective to improve the subframe or bush at 57 Hz.

Therefore, it was summarized as:

1. Contact forces better represented the characteristics of the combined system compared to the existing frequency response function; thus, it was used as an indicator for the selection of subsystems combination.
2. Contact forces were used except for subsystems containing the final response. Contact force was the force generated between connected subsystems; hence, if the final response point was not connected, the force was obtained using transmitted force.
3. It was possible to identify which transfer path was a problem in the subsystems using the force transmissibility ratio. So, the force transmissibility ratio was used as a basis for quantitative judgment on which subsystem to improve.

Force transmissibility can determine the subsystem to be improved, so if there are multiple subsystems, it can be utilized to find improved subsystem combinations through characteristic calculation for multiple combinations.

In this section, the strategy to find a combination of subsystems and targets for improvement when predicting characteristics using substructuring was described. The next section presents a methodology for how to manage subsystem in the database.

### Method of managing the characteristics of subsystems

It was difficult to directly use the frequency response function or contact force to manage the subsystems characteristics within the database. The frequency response function of the subsystem was measured in the state where the boundary condition was the free-end. This was an advantage in measurement; however, an error occurred because the boundary condition was not reflected during the connection. Accordingly, to manage the characteristics of subsystems in the database, an indicator representing the characteristics of subsystems itself was required. In other words, it should be able to be directly used when connecting to other subsystems.

Blocked force, which has been widely studied recently, is a force independent of the characteristics of the system to which parts are connected and is not subordinate to the interrelationship between sources and receivers. It is used to predict the response using FBS when the system to which the excitation source is connected changes in the experiment. If the system to which the excitation source is connected changes, the characteristics of the connection part between the new system and the existing system are different from the existing connection characteristics, resulting in an error. If a sensor is mounted on the connection part of the existing system to measure the force, the connection characteristics change, making it difficult to obtain accurate data. A method of measuring the force such that the force transmitted by the system connected with the excitation source has characteristics independent of other substructures to improve this was the blocked force.

**Figure 8:**
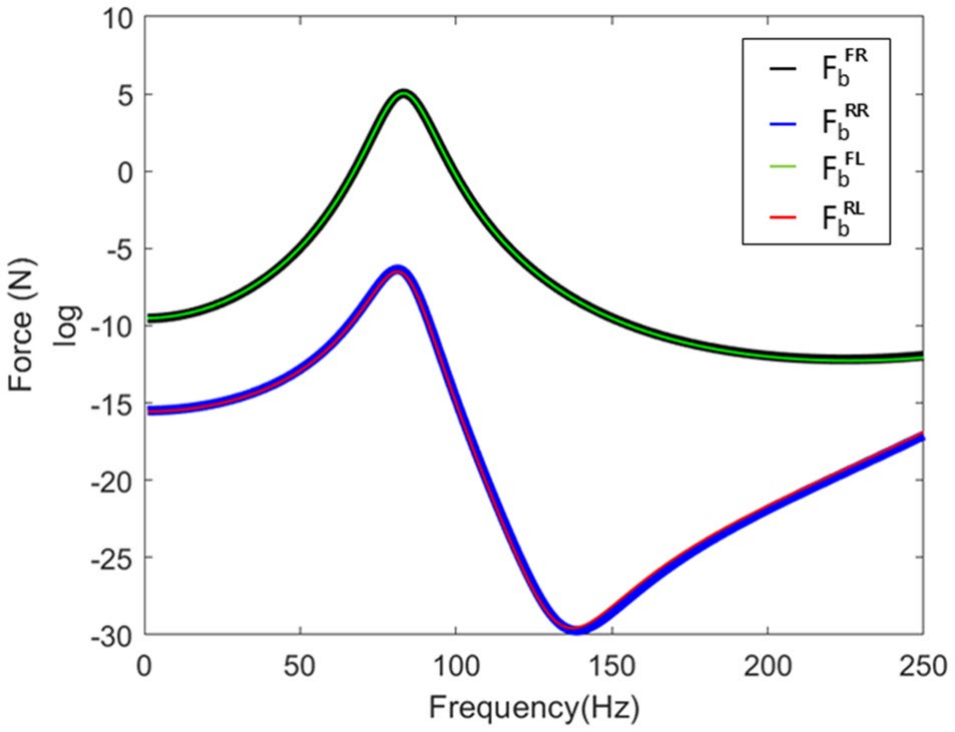
The blocked force generated at the connection part.

To calculate the blocked force, the measurement subsystem must be clamped. Theoretically, to derive results, a rigid boundary must be connected to the subsystem. In other words, the theoretical modeling method calculated the blocked force by modeling a large mass at the measurement position. Figure 8 presents the blocked force of the subframe.

**Figure 9:**
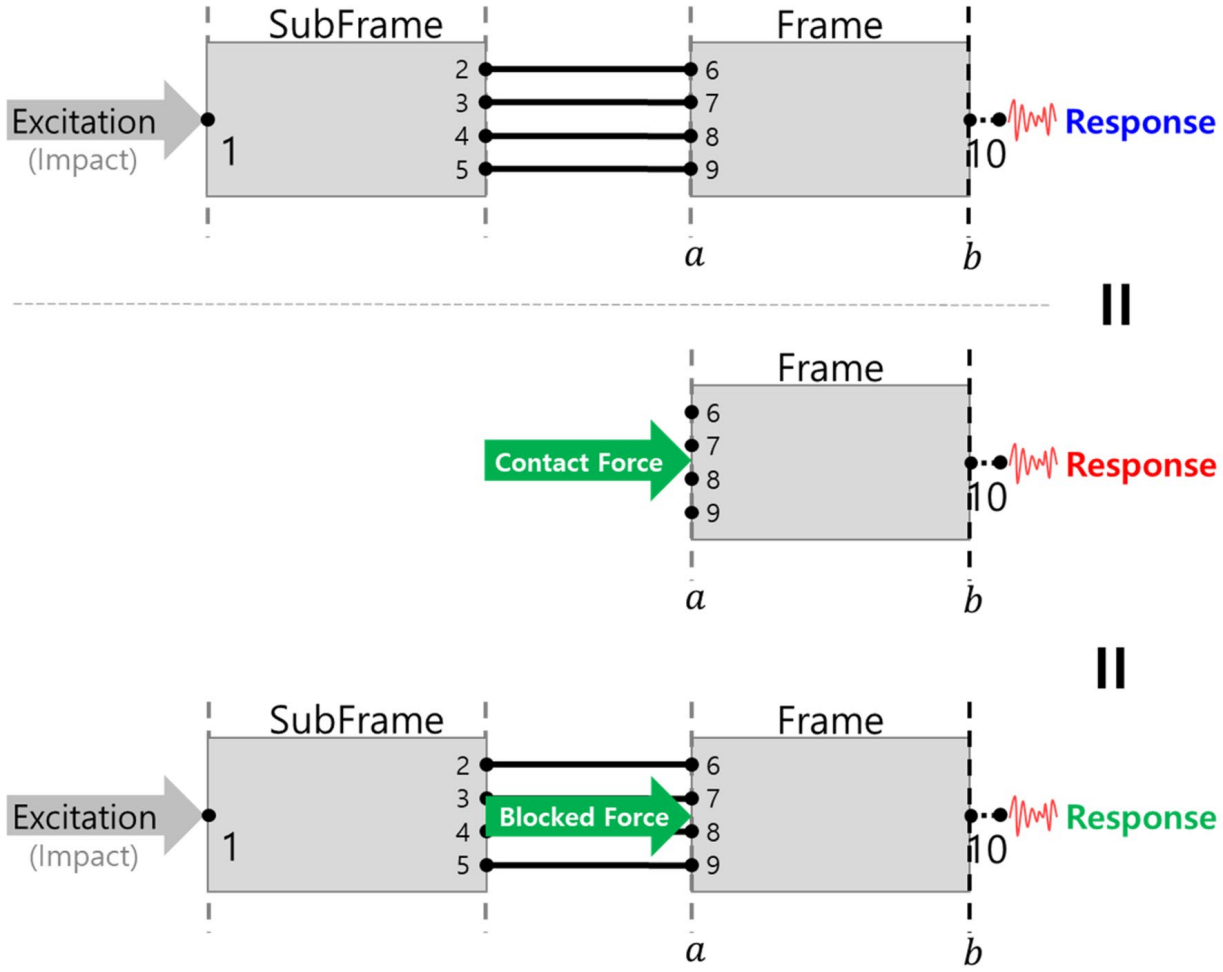
Response prediction using contact force and blocked force.

**Figure 10:**
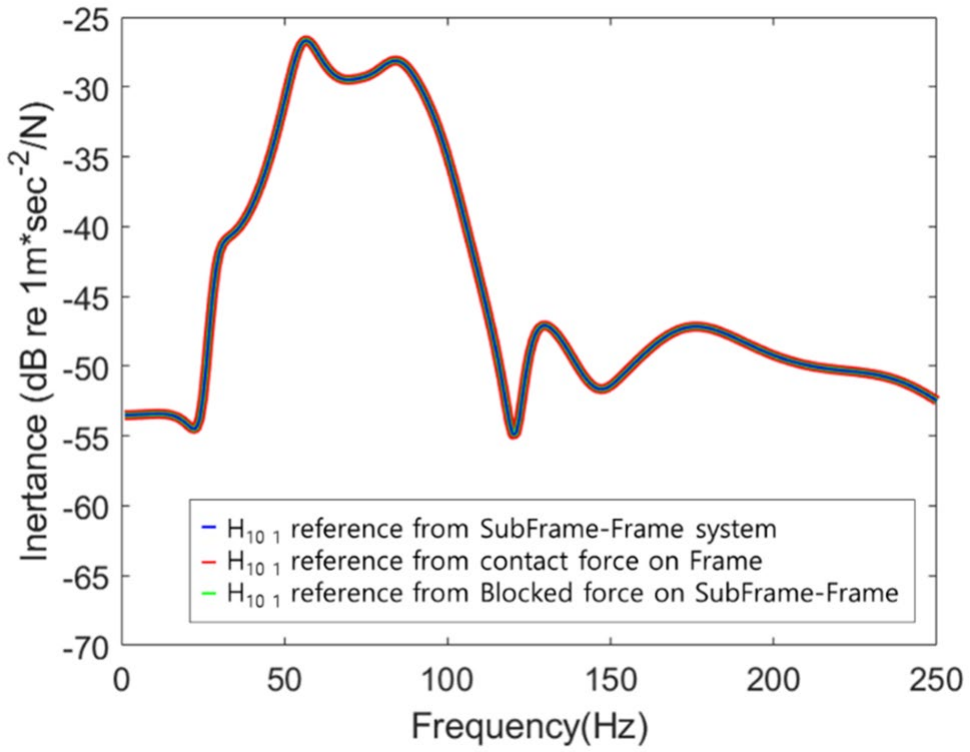
Response of the simplified structure.

This blocked force was connected to a new frame to obtain a response, and the previously calculated contact force was applied to a new frame to obtain a response. This was simply schematized (Fig. 9), and Fig. 10 shows the results.

**Figure 11:**
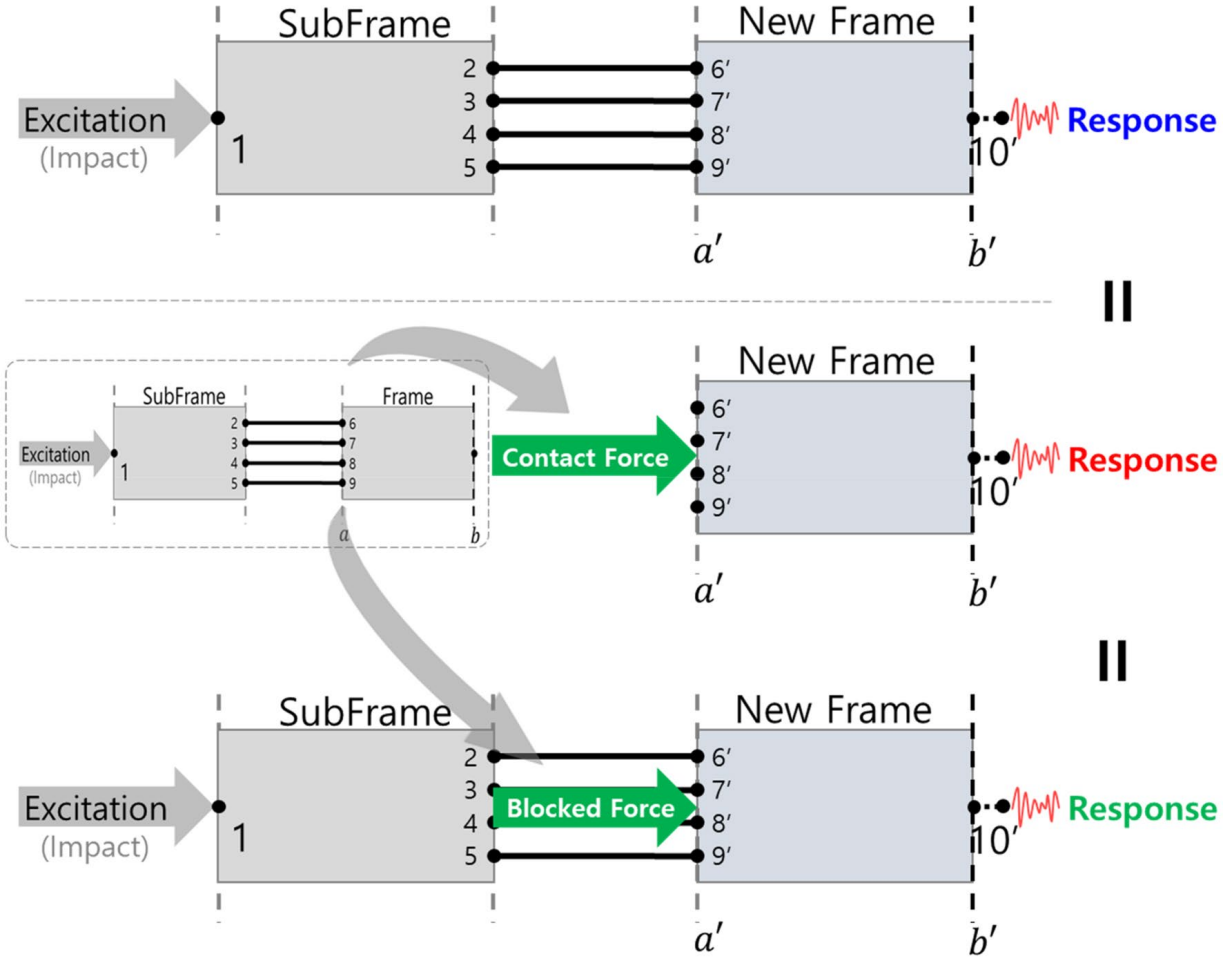
Schematic diagram of the response to the application of a new frame.

Since the contact force was a force generated at the connection point of the frame, it was made to excite the connection point of the new frame, and because the blocked force was not related to the connection characteristics of the frame, it excited the connection part of the subframe-frame structure. Based on the simulation results, it was identified that all three results were the same. It was applied to a new simplified system in which a new frame and an existing subframe were connected (Fig. 11).

**Figure 12:**
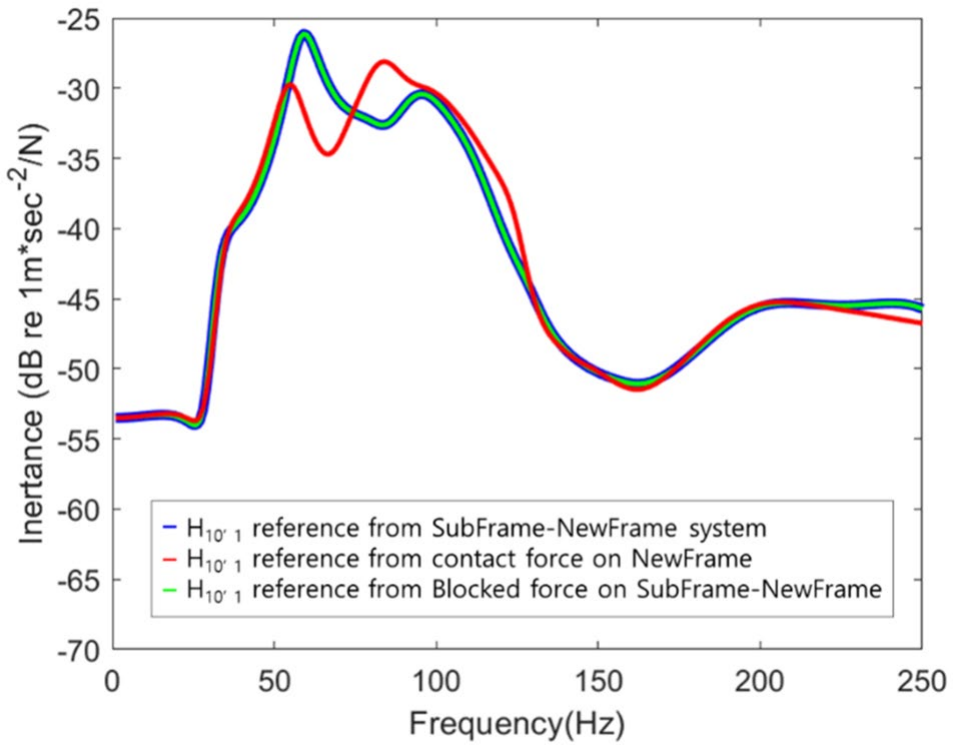
Prediction of responses according to frame changes using contact force and blocked force.

It was identified that the blocked force was predicted even after the change of the receiver’s subsystem because it was independent of the connection characteristics (Fig. 12). On the contrary, the contact force was difficult to apply to a new subsystem because it was related to the connection characteristics of the existing simplified subsystem. Therefore, when the parts changed, it was necessary to recalculate them.

Through this, it was identified that the characteristics of the system change due to the connection part, and this was used when combining subsystem. Moreover, it was found that the blocked force could be used as a subsystem management indicator because it was independent of the connection characteristics.

Therefore, it was summarized as:

1. It was necessary to manage the characteristics of subsystem combine subsystem based on the database, and it was difficult to use the frequency response function or contact force because the characteristics changed depending on the connection parts. However, the blocked force exhibited characteristics independent of connection; thus, it was suitable for the characteristics management of a subsystem within the database.
2. When combining subsystems using the contact force, the subsystems must be connected under free-free conditions. In contrast, the blocked force obtained a response by applying it to the connection part.

### Prediction of characteristics of subsystem with artificial neural network

There were many previously developed data for each subsystem of vehicles in modular platform strategy. Therefore, when predicting characteristics in the vehicle development stage, it was possible to efficiently improve product development time and effort if it was possible to perform prediction of new improvement plans in advance based on the previously data. An ANN algorithm was used as a method of training and predicting data previously developed. Artificial neural network algorithms are algorithms aimed at realizing complex information processing capabilities intelligently processed by humans through machines by imitating the human brain structure and information processing methods performed in the brain. To perform the ANN algorithm, modeling of each neuron must be performed first, followed by modeling of the structure of the neural network created by connecting neurons.

**Figure 13:**
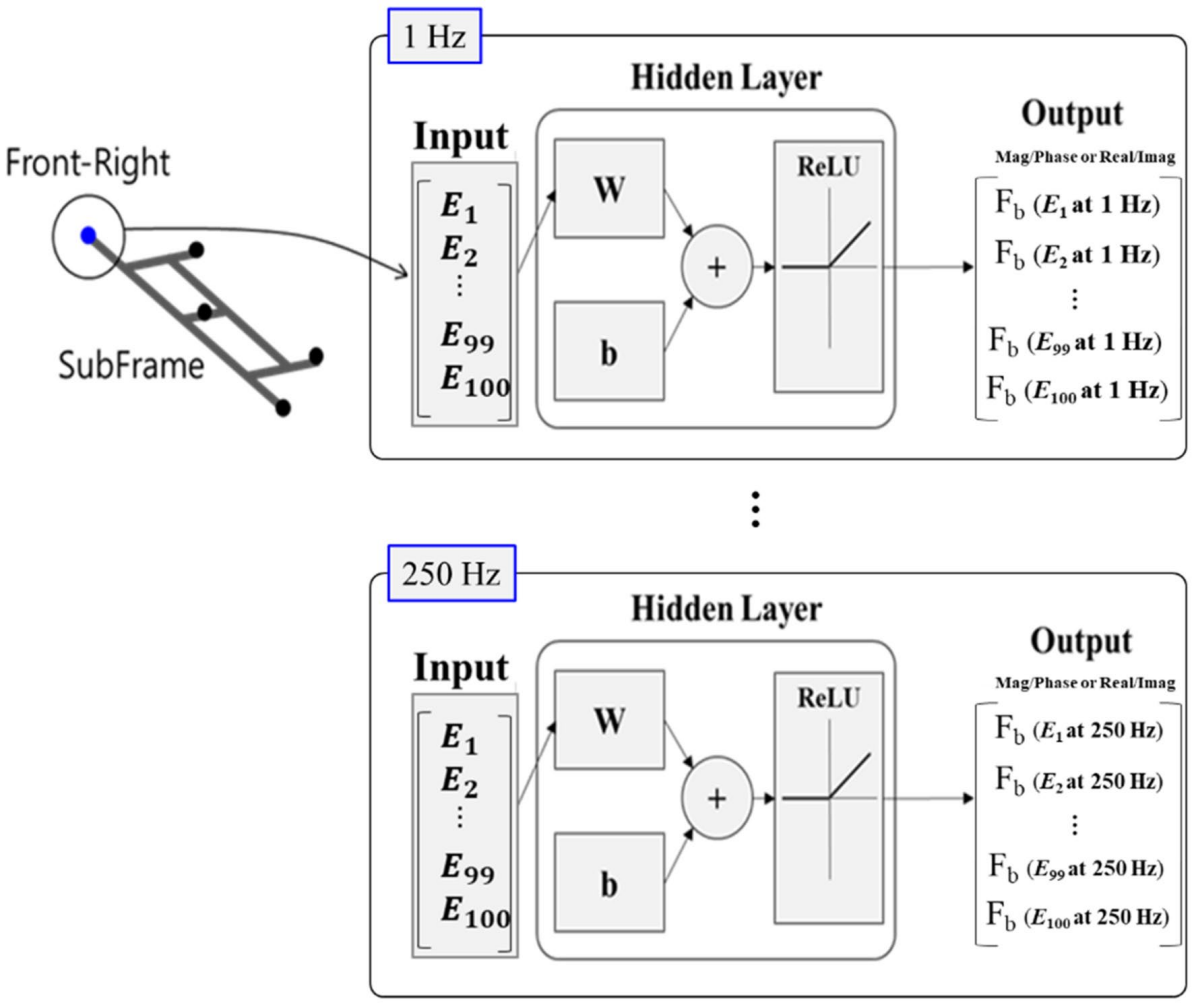
ANN training according to the change of Young's modulus of the subframe.

Here, subframe was selected as the part to be predicted. Moreover, the input of the neural network model was set to be Young's modulus, one of the design variables, and the output was set to predict the blocked force, an indicator of subsystem characteristics management (Fig. 13). Young's Modulus was changed assuming that the front-right connection part of the subframe was to be improved. The Young’s modulus (210GPa) of subframe at the connection location is adjusted to a ratio of 0.5 to 1.49 times (0.01 increments) to construct the input data. Therefore, the set of input data consists of *E*_1_ (105 * 0.5 GPa), *E*_2_ (105 * 0.51 GPa), *E*_3_ (105 * 0.52 GPa), $\ldots$, and *E*_100_ (105 * 1.49 GPa). The blocked force to be predicted was set in the range of 1 to 250 Hz at 1 Hz intervals. In terms of the expression methods of blocked force, the two methods of Magnitude/Phase and Real/Imaginary part were compared. Therefore, the output is configured to predict the Magnitude/Phase and Real/Imaginary part of the Blocked force every hertz, and the prediction was repeatedly performed 250 times every hertz to predict the 1 to 250 Hz band. K-fold cross validation was used for data training. K-fold cross validation is a statistical analysis method in which the collected samples are cross-validated by making k-folds, and it can improve accuracy in cases where the total number of data is small. In addition, the early stop was applied to prevent overfitting. For the configuration of the neural network algorithm, the fitnet of Matlab, a commercial software, was used, and Bayesian regularization backpropagation was used for the training function. Training parameters consisted of the following: Marquardt adjustment parameter was 0.005, decrease/increase factor for Marquardt adjustment parameter was 0.1 and 10, respectively and minimum performance gradient was 1e−7.

The number of nodes in Hidden layers was set differently for magnitude/phase and real/imaginary part of blocked force data, respectively. In predictions using Magnitude/Phase, hidden layers consist of two layers, each consisting of 10 and 8 nodes. Similarly, two hidden layers consist of 8 and 5 nodes. For each hertz, the input data was 1 × 100 vectors and the output data was composed of 1 × 100 matrices. That is, since the frequency band of interest is 1 to 250 Hz, the 250 by 100 matrix becomes the final output. The number of datasets is 100, which are randomly divided into training sets and test sets at an 8:2 ratio. Data preprocessing of input/output dataset was performed using MinMax normalization method.

**Figure 14:**
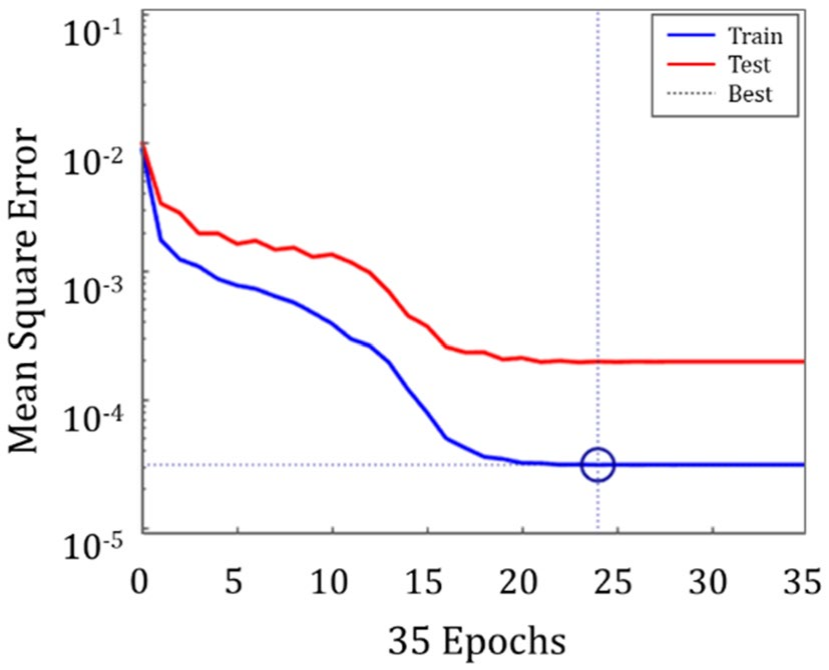
Mean squared error versus number of epochs for training and test data.

An example of mean squared error versus number of epochs for real/imaginary part of blocked force data is shown in Fig. 14.

Based on the training results, the mean square error for the pretrained model is tabulated in Table 2.

**Table 2:** Training results.

	Input data form: magnitude/phase (%)	Input data form: real/imag. part (%)
Mean absolute error of the pretrained model	8.0782	1.2442
Test set error	10.2452	1.9522

**Figure 15:**
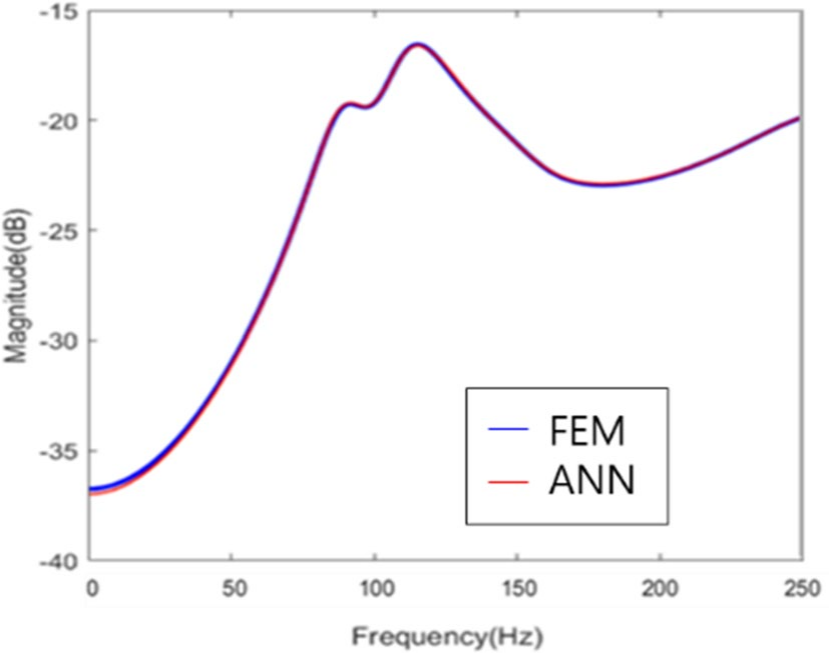
Blocked force prediction results.

On the basis of the results, it was found that training the real/imaginary part predicted more accurate results than training the magnitude and phase of the blocked force. The representative blocked force prediction result is expressed in Fig. 15.

Based on the prediction results, it was identified that the magnitude of the blocked force was predicted with the error within 2.0% on average for the data, indicating that the results were similar to the results of analyzing the actual FEM. Therefore, if there were pre-trained model built with ANN, the blocked force of a new substructure could be easily predicted when developing another vehicle, and the characteristics of the system could be identified in advance; thus, an efficient development could be conducted.

**Figure 16:**
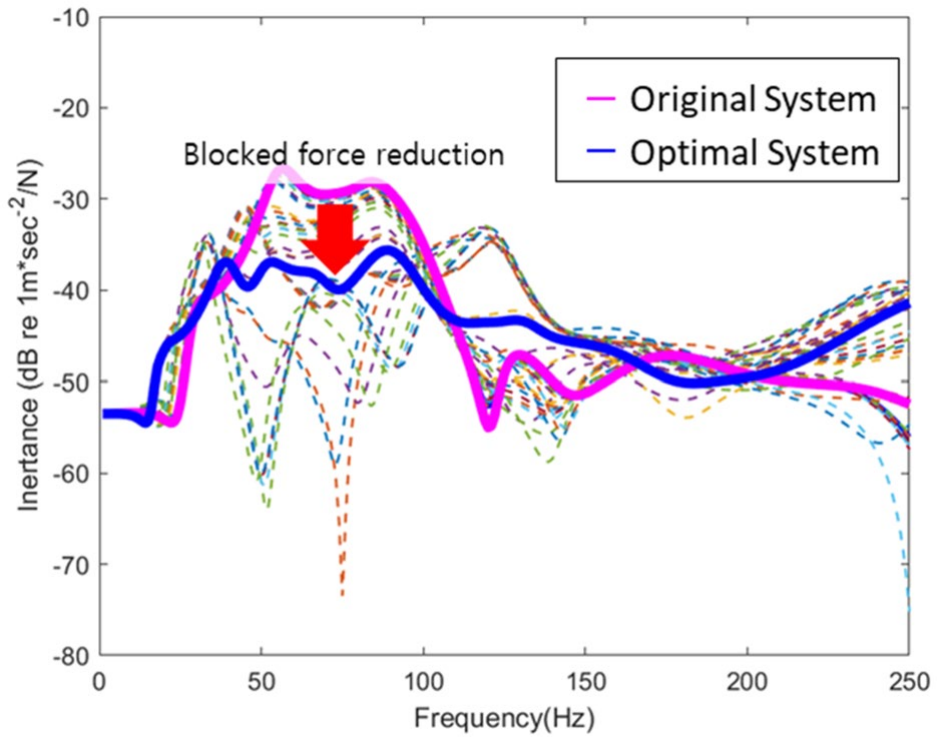
Improvement of the response of the simplified structure.

Finally, the lowest root sum square of blocked force at the interval of interest (1–250 Hz) was selected and the response of simplified structure when the blocked force of the optimal subframe was connected to the frame was presented in Fig. 16. Based on the results, it was confirmed that the blocked force of the improved subsystems in the database was reduced, in turn, reducing the final response. To this end, data not present in the database could be predicted using ANN and used to preemptively reduce the response in the concept stage of a vehicle development process.

## Conclusions

In this paper, substructuring and ANN based VPD method for designing at the concept stage was proposed. Vibration characteristics of the system according to the combination of subsystems were predicted through a dynamics-based substructuring technique. A simple vehicle frame structure was modeled and verified using an LM-FBS. Through this, it was confirmed that it was difficult to identify the characteristics of the system through the frequency response function of the subsystems when determining improved subsystems or combinations of subsystems. Because the frequency response function of the subsystems didn’t reflected the connection characteristics, the contact force, an indicator that reflected the connection characteristics, was used as an indicator related to the combination of subsystems. Moreover, the transmitted force was introduced at the response point. Force transmissibility was used to select improved subsystems. This Force transmissibility methodology has the advantage of being able to clearly compare the transmission of forces between subsystems rather than the existing contribution evaluation or transfer path analysis, making it easier to identify the improvement subsystem. For subsystem management in the database, a blocked force that did not reflect the connection characteristics was introduced. It was confirmed through simulation that a blocked force independent of the connection characteristics was essential when applying the modular platform strategy because various combinations were made between subsystems.

Finally, when subsystems in the database were subject to improvement, a blocked force that was not in the database was predicted using the ANN. In terms of the data representation methods for training the blocked force, it was confirmed that the method of expressing it with real and imaginary parts showed a higher accuracy than the method of expressing it with magnitude and phase. Finally, it was confirmed that the final response was improved by connecting the improved subframe with the original substructure. Such a method of improving subsystem using an ANN had the advantage in that it took less time and effort than the existing method using the finite element method; thus, it could be applied at the concept stage of vehicle development. Finally, it was confirmed that the response was reduced by connecting the optimal subframe to the original system, and a vehicle development process using a database at the concept stage was proposed.

## Data Availability

The datasets used and/or analysed during the current study available from the corresponding author on reasonable request.

## References

[1] Singer DJ, Doerry N, Buckley ME (2009). What is set-based design?. Nav. Eng. J..

[2] Specking E (2018). Literature review: Exploring the role of set-based design in trade-off analytics. Nav. Eng. J..

[3] Liker JK, Ettlie JE, Campbell JC (1995). Engineered in Japan: Japanese technology-management practices.

[4] Durward SI, Ward AC, Liker JK (1999). Toyota’s principles of set-based concurrent engineering. MIT Sloan Manag. Rev..

[5] Stocklosa, A. Cowa-TNGA! Toyota’s new modular platform reaching production this year. Car and Driver https://www.caranddriver.com/news/a15357286/cowa-tnga-toyotas-new-modular-platform-reaching-production-this-year/ (2015)

[6] Lampón JF, Cabanelas P, Benito JG (2015). The impact of implementation of a modular platform strategy in automobile manufacturing networks. GEN Gov. Econ. Res. Netw..

[7] Zhang, W., Roller, D., Chen, W. & Zou, H. *Perspectives from Europe and Asia on engineering design and manufacture* (eds Yan, X. T. et al.) 79–88 (Springer Dordrecht, 2004).

[8] Shabana AA (2019). Integration of computer-aided design and analysis: Application to multibody vehicle systems. Int. J. Vehicle Perform..

[9] Kleark DD (2009). Dynamic Response Characterization of Complex Systems Through Operational Identification and Dynamic Substructuring.

[10] Rixen DJ, Van der Valk PLC (2013). An impulse based substructuring approach for impact analysis and load case simulations. J. Sound Vib..

[11] Voormeeren SN (2012). Dynamic Substructuring Methodologies for Integrated Dynamic Analysis of Wind Turbines.

[12] Mahmoudi AE, Rixen DJ, Meyer CH (2020). Comparison of different approaches to include connection elements into frequency-based substructuring. Exp. Tech..

[13] Chen K, Herrin DW (2020). Numerical and Experimental Studies of Blocked Force Determination on an Offset Interface for Plate and Shell Structures and Duct Acoustic Systems.

[14] Fox RL, Kapoor MP (1968). Rates of change of eigenvalues and eigenvectors. AIAA J..

[15] Rogers LC (1970). Derivatives of eigenvalues and eigenvectors. AIAA J..

[16] Garg S (1973). Derivatives of eigensolutions for a general matrix. AIAA J..

[17] Nelson RB (1976). Simplified calculation of eigenvector derivatives. AIAA J..

[18] Lim KB, Junkins JL, Wang BP (1987). Re-examination of eigenvector derivatives. J. Guid. Control Dyn..

[19] Pandey PC, Barari SV (1992). Multilayer perception in damage detection of bridges structures. Comput. Struct.

[20] Atalla MJ, Inman DJ (1998). On model updating using neural networks. Mech. Syst. Signal. Process.

[21] Levin RI, Lieven NAJ, Lowenberg MH (2000). Measuring and improving neural network generalization for model updating. J. Sound Vib..

[22] Hossain MS, Ong ZC, Ismail Z, Noroozi S, Khoo SY (2017). Artificial neural networks for vibration based inverse parametric identifications: A review. Appl. Soft Comput..

[23] Lage YE, Neves MM, Maia NMM, Tcherniak D (2014). Force transmissibility versus displacement transmissibility. J. Sound Vib..

